# ApoE deficiency exacerbates the development and sustainment of a semi-chronic K/BxN serum transfer-induced arthritis model

**DOI:** 10.1186/s12967-016-0912-y

**Published:** 2016-06-10

**Authors:** Amy M. Archer, Rana Saber, Shawn Rose, Alexander Shaffer, Alexander V. Misharin, FuNien Tsai, G. Kenneth Haines III, Salina Dominguez, Mesut Eren, Douglas E. Vaughan, Carla M. Cuda, Harris Perlman

**Affiliations:** Division of Rheumatology, Department of Medicine, Northwestern University Feinberg School of Medicine, 240 East Huron Street, McGaw M338, Chicago, IL 60611 USA; Immunoscience Exploratory Clinical and Translational Research, Bristol-Myers Squibb, Lawrenceville, NJ USA; Division of Pulmonary and Critical Care, Department of Medicine, Northwestern University Feinberg School of Medicine, Chicago, IL USA; Department of Pathology, Mount Sinai Hospital, New York, NY USA; Division of Cardiology, Cardiovascular Research Institute, Northwestern University Feinberg School of Medicine, Chicago, IL USA

**Keywords:** Arthritis, Cholesterol, Inflammation, Animal models of human disease

## Abstract

**Background:**

The risk for developing cardiovascular disease is greater in patients with rheumatoid arthritis (RA) than in the general population. While patients with RA also have dyslipidemia, the impact of dyslipidemia on the severity of inflammatory arthritis and associated cardiovascular disease is unclear. Currently, there are conflicting results regarding arthritis incidence in apolipoprotein E (ApoE) deficient mice, which spontaneously exhibit both hyperlipidemia and atherosclerosis. Here, we utilize a distinct approach to investigate the contribution of a hyperlipidemic environment on the development of arthritis and atherosclerosis in mice lacking ApoE.

**Methods:**

K/BxN serum transfer-induced arthritis (STIA) was assessed in C57BL/6 (control) and ApoE^−/−^ mice using clinical indices and immunohistochemical staining. Ankle synoviums were processed for flow cytometry. Aortic atherosclerosis was quantitated using Sudan IV staining. Serum cholesterol and cytokine levels were determined via enzymatic and luminex bead-based assays, respectively.

**Results:**

ApoE^−/−^ mice developed a sustained and enhanced semi-chronic inflammatory arthritis as compared to control mice. ApoE^−/−^ mice had increased numbers of foamy macrophages, enhanced joint inflammation and amplified collagen deposition versus controls. The presence of arthritis did not exacerbate serum cholesterol levels or significantly augment the level of atherosclerosis in ApoE^−/−^ mice. However, arthritic ApoE^−/−^ mice exhibited a marked elevation of IL-6 as compared to non-arthritic ApoE^−/−^ mice and arthritic C57BL/6 mice.

**Conclusions:**

Loss of ApoE potentiates a semi-chronic inflammatory arthritis. This heightened inflammatory response was associated with an increase in circulating IL-6 and in the number of foamy macrophages within the joint. Moreover, the foamy macrophages within the arthritic joint are reminiscent of those within unstable atherosclerotic lesions and suggest a pathologic role for foamy macrophages in propagating arthritis.

**Electronic supplementary material:**

The online version of this article (doi:10.1186/s12967-016-0912-y) contains supplementary material, which is available to authorized users.

## Background

Rheumatoid arthritis (RA) is a chronic, systemic, autoimmune disease associated with significant disability and increased mortality [1]. Life expectancy is reduced in patients with RA, largely due to the development of premature cardiovascular disease [2, 3]. Patients with RA have roughly a 50–60 % increase in the risk of death from cardiovascular causes compared to the general population [4]. This elevated cardiovascular risk is independent of traditional risk factors and may be due to increased systemic inflammation in RA [3].

Atherosclerotic disease is a major cause of cardiovascular morbidity and mortality. One of the primary determinants of the propensity of atherosclerotic plaques to rupture and subsequently cause vascular occlusion is the stability of the foamy macrophages within the plaques [5]. Foamy macrophages are formed through the uptake of oxidized LDL through scavenger receptors, such as CD36. When foamy macrophages become saturated with oxidized LDL cell death can occur, which results in the release of proinflammatory cellular contents and formation of a necrotic core within the atherosclerotic plaque [5]. The understanding of this process has been facilitated by the use of ApoE knockout mice [6, 7] since functional mutations in ApoE lead to elevations in serum cholesterol levels and premature atherosclerosis in both mice and humans [8, 9].

The role that dyslipidemia plays in the development of atherosclerosis in RA is highly controversial. Numerous studies have shown that patients with RA have reduced total cholesterol (TC) levels compared to controls, which may be attributed to the increased inflammatory burden present in RA [10]. Surprisingly, decreases in TC are associated with adverse cardiovascular outcomes in patients with RA [11]. However, the atheroprotective effects of high-density lipoprotein (HDL) may be dampened in RA, as patients with more active disease exhibit reduced HDL-mediated cholesterol efflux as compared to patients with quiescent disease [12].

Conversely, the impact of the presence of hyperlipidemia on the development of inflammatory arthritis is also unclear. The collagen-induced arthritis (CIA) model [13] has previously been utilized to examine the development of arthritis and atherosclerosis in ApoE^−/−^ mice, but has led to conflicting results. One group found that ApoE^−/−^ mice were resistant to CIA [14], while another group showed that ApoE^−/−^ mice developed more severe disease [15]. Thus the uncertainty of the role that dyslipidemia plays in the progression of arthritis in RA and whether the changes in lipid profile associated with inflammatory arthritis can alter the progression of atherosclerosis in a hyperlipidemic setting further underscores the critical need to utilize murine models of altered cholesterol metabolism to better understand these processes.

Here, we examined the development of arthritis in ApoE^−/−^ mice compared to controls using a semi-chronic K/BxN serum transfer-induced arthritis (STIA) model [16]. The STIA model mimics the effector phase of RA-like disease and does not require lymphocytes. ApoE^−/−^ mice developed enhanced semi-chronic inflammatory arthritis as compared to control mice. Elevated foamy macrophage numbers were observed in the synovial lining and extra-articular tissue from ApoE^−/−^ mice, but not in controls. Moreover, arthritic ApoE^−/−^ mice secreted increased levels of circulating pro-inflammatory IL-6. However, the degree of arthritis failed to significantly impact dyslipidemia or atherosclerotic disease burden in ApoE^−/−^ mice on a high fat diet. These data suggest that a dyslipidemic environment can alter the progression of inflammatory arthritis during the effector phase of disease. Further, these data support a potential pathogenic role for foamy macrophages not only in atherosclerotic lesions, but also within the arthritic joint.

## Methods

### Animals

For generation of arthritogenic serum, KRN mice were kindly provided by Drs. Diane Mathis and Christophe Benoist, and non-obese diabetic mice were purchased from Taconic, Germantown, NY. Male and female wild-type control mice (C57BL/6, Jackson Laboratory, Bar Harbor, ME) and ApoE^−/−^ mice, which spontaneously exhibit both hyperlipidemia and atherosclerosis (Jackson Laboratory—B6.129P2-Apoe^tm1Unc^/J stock number 002052), were purchased from commercial vendors. Genotyping was confirmed by Transnetyx (Memphis, TN). Mice were maintained on chow until 8 weeks of age and then switched to Harlan Teklad Western diet TD.94059 (Harlan Technologies, Houston, TX) containing 15.8 % fat and 1.25 % cholesterol. Animals were bred and maintained in a barrier facility within the Center for Comparative Medicine and were approved by the Northwestern University IACUC.

### Scoring and induction of arthritis

For acute K/BxN serum transfer-induced arthritis (STIA), animals received a single injection of 50 μl of K/BxN serum at 12 weeks of age as previously described [17–19]. STIA involves the passive transfer of arthritogenic anti-glucose-6-phosphate isomerase antibodies. The arthritis that ensues in these mice has almost 100 % penetrance and the mice develop maximum arthritis within 1 week after each injection of K/BxN serum. Arthritis severity was determined via the change in thickness of the hind paw ankle joints and a clinical score (total = 12) for all four paws, where 0 = normal, 1 = swollen wrist, 2 = swelling extending to dorsal paw, 3 = swelling extending to the digits. Acute arthritis severity was assessed every other day via clinical score for 2 weeks. Semi-chronic K/BxN STIA was elicited in ApoE^−/−^ and control (C57BL/6) mice using 50 μl of K/BxN serum every 2–3 weeks as previously described [17, 20]. Animals were scored weekly for arthritis severity.

### Tissue preparation and flow cytometry

Peripheral blood was harvested by cardiac puncture, followed by ventricular perfusion with 20 ml of PBS. Aortas were excised, fixed in 4 % paraformaldehyde and stained with Sudan IV (Sigma-Aldrich, St. Louis, MO). Ankles were either fixed in 10 % formalin, decalcified and then paraffin embedded prior to immunohistochemistry or processed for flow cytometry as previously described [17]. Data were acquired on BD LSR II flow cytometer. Additional file 1 lists antibodies, clones, fluorochromes and manufacturers used for all flow cytometric studies. The following dyes were utilized: fixable Viability Dye eFluor 506 from eBioscience and BoDIPY 493/503 from Molecular Probes. Compensation and analysis of the flow cytometry data were performed using FlowJo software (TreeStar).

### Immunohistochemistry

Paraffin-embedded ankle sections were stained with haematoxylin and eosin, picrosirius red, rat anti-mouse F4/80 (clone BM8, Caltag) antibody or CD45 (Caltag) antibody and were scored for severity of disease by a pathologist (GKH) blinded to the conditions as previously described [20–23]. Imaging was performed using an Olympus DP40 microscope (Tokyo, Japan) equipped with a DP71 camera or a Zeiss upright AXIO (TissueGnostics GmbH) with a polarizing filter at the Northwestern Cell Imaging Facility (supported by NCI CCSG P30 CA060553 awarded to Robert H Lurie Comprehensive Cancer Center). Acquisition and analysis of picrosirius red staining utilized Tissue FAXS and TissueQuest. The picrosirius red quantification represents the sum of trans mean intensity for each tissue section.

### Serum analysis

Serum TC, low-density lipoprotein/very low-density lipoprotein (LDL/vLDL) and HDL cholesterol levels were measured using enzymatic assays in accordance with the manufacturer’s instructions (AbCam, Cambridge, MA). Serum cytokine and chemokine levels were assessed using Luminex (Austin, TX) Panomics 16-plex assays (Affymetrix, Santa Clara, CA) according to the manufacturer’s instructions.

### Evaluation of atherosclerosis

Atherosclerosis was quantified by computer-assisted morphometric analysis (Cell sense and ImagePro 6.3 software) of whole aortic (aortic root to iliac bifurcation) specimens. Results were expressed as the percentage of Sudan IV-stained lesion area (calculated by Sudan IV stained area/total aortic area × 100).

### Statistical analysis

GraphPad Prism 5.0 Software (GraphPad Software, San Diego CA, USA) was utilized for all statistical analyses. Two-way ANOVA with Bonferroni posttests were used for arthritis analyses. Mann–Whitney U tests were used for histologic scoring, Sudan IV staining, levels of cytokines and chemokines, flow cytometry analysis, TC, LDL/vLDL and HDL levels among groups of mice. Statistical significance was established at p < 0.05.

## Results

### ApoE^−/−^ mice exhibit greater severity of semi-chronic arthritis than control mice

There are conflicting data concerning the effect of ApoE deficiency on the development of inflammatory arthritis in the CIA model [14, 15]. Here we employed a different model of RA-like disease, the STIA model, which mimics the effector phase of RA and does not require the adaptive immune response. When the traditional STIA model was utilized (i.e., single injection of serum), we observed no difference in arthritis severity between ApoE^−/−^ mice and C57BL/6 mice over a 2-week period (Additional file 2). However, this acute model represents a shorter time frame than the CIA model and does not provide adequate time for atherosclerotic plaques to develop. Therefore we employed a modified semi-chronic version of the STIA model, where animals were injected with K/BxN serum every 2 weeks starting at 8 weeks of age and the development of arthritis was scored weekly over a 2–4 month period [24].

**Fig. 1:**
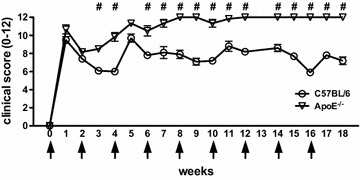
Semi-chronic arthritis is more severe in ApoE^−/−^ mice compared to C57BL/6 mice. C57BL/6 (control) and ApoE^−/−^ mice (n = 4–5 mice/group) were fed chow until 8 weeks of age, at which time they were fed a Western diet for an additional 2–4 months. Animals received serial injections of K/BxN serum every 2–3 weeks, beginning at 8 weeks of age. Arthritis severity was assessed weekly via clinical score. Data are represented as mean ± SEM. *Asterisk* denotes statistically significant differences: ^#^p < 0.001 for ApoE^−/−^ as compared to control mice. *Arrows* indicate time of injections

**Fig. 2:**
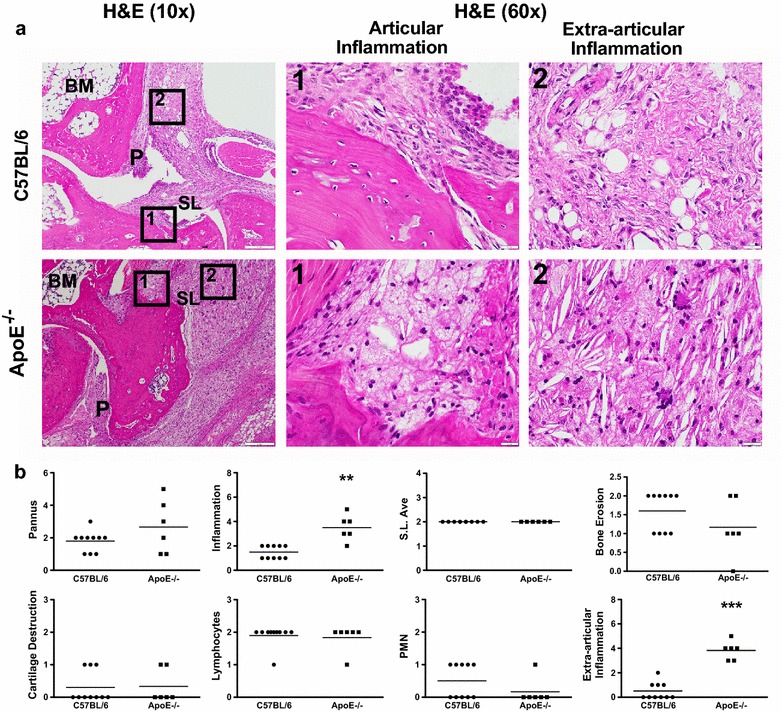
Increased articular and extra-articular inflammation is present in ApoE^−/−^ mice. **a** H&E staining of arthritic ankle joints from C57BL/6 (control, n = 10) and ApoE^−/−^ (n = 6) mice. *Boxes* *1* and *2* demarcate articular and extra-articular areas of high magnification (×60), respectively. **b** Histopathological scores for pannus formation, inflammation, synovial lining average, bone erosion, cartilage destruction, lymphocytes, polymorphonuclear cells and extra-articular inflammation on ankle joints from above. Data are represented as mean ± SEM. *Asterisk* denotes statistically significant differences: **p < 0.01, ***p < 0.001. *BM* bone marrow, *SL* synovial lining, *P* pannus

**Fig. 3:**
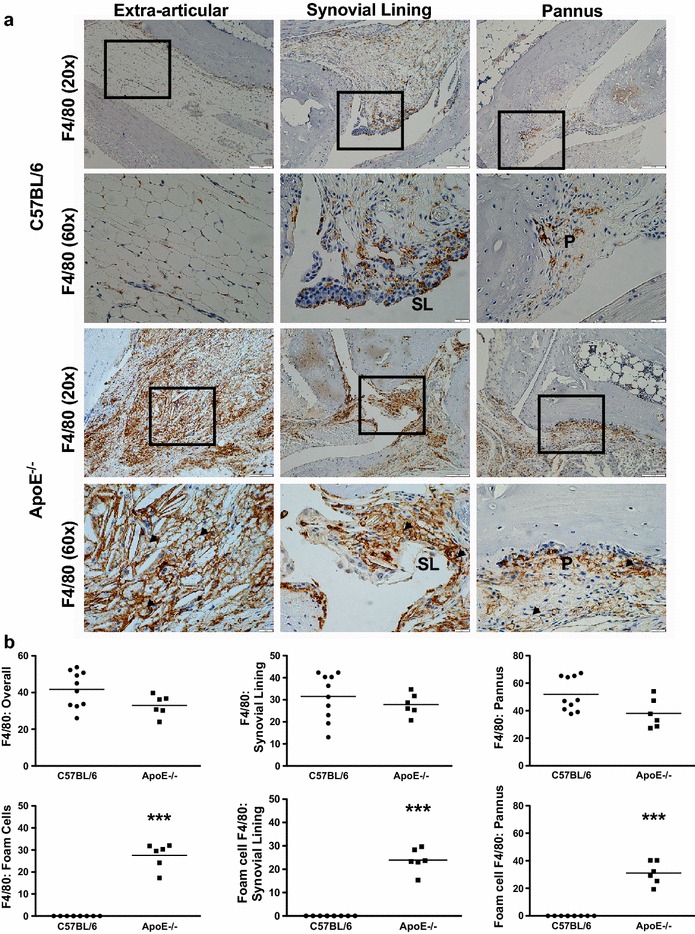
Arthritic ApoE^−/−^ mice have increased F4/80^+^ foam cells. **a** Photomicrographs of F4/80-stained extra-articular tissue, synovial lining and pannus of arthritic C57BL/6 (control, n = 10) and ApoE^−/−^ (n = 6) mice. *Squares* delineate area of higher magnification shown below the respective image. **b** Immunohistopathological scores for F4/80^+^ total cells, F4/80^+^ cells within synovial lining and F4/80^+^ cells within the pannus from control and ApoE^−/−^mice. F4/80^+^ foam cells within each of these categories are subsequently quantified from the mice described above. Data are represented as mean ± SEM. *Asterisk* denotes statistically significant differences: ***p < 0.001. *SL* synovial lining, *P* pannus, *arrowheads* foamy macrophages

**Fig. 4:**
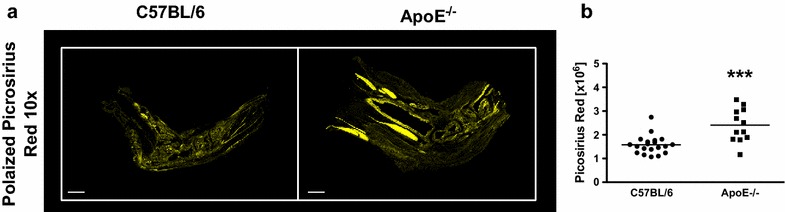
Arthritic ApoE^−/−^ mice have increased levels of collagen. **a** Picrosirius red-positive tissue sections of arthritic C57BL/6 (control, n = 10) and ApoE^−/−^ (n = 6) mice. Picrosirius red staining is pseudocolored *yellow*. **b** Quantification of picrosirius red staining on tissue sections (2 sections/mouse) described above. Data are represented as mean ± SEM. *Asterisk* denotes statistically significant differences: ***p < 0.001

Throughout the development of semi-chronic arthritis ApoE^−/−^ mice demonstrated significantly (p < 0.001) more severe inflammatory arthritis as compared to C57BL/6 mice (Fig. 1). The clinical scores were also associated with the presence of histologically assessed inflammation. Ankle joint histologic severity scores of inflammation were elevated 2.3-fold in arthritic ApoE^−/−^ mice compared to C57BL/6 mice (Fig. 2a, b). Additionally, extra-articular inflammation was increased 7.7-fold in arthritic ApoE^−/−^ mice compared to C57BL/6 mice (Fig. 2a, b). The histologic appearance of the extra-articular inflammation resembled that of a vulnerable atherosclerotic plaque, which is unstable and thus has an increased risk of progressing from a non-obstructive lesion to a vascular occlusion [25]. Although there were not significant differences in the total number of F4/80^+^ macrophages or in the number of F4/80^+^ macrophages within the synovial lining or pannus (Fig. 3a, b), when we focused on the subset of F4/80^+^ macrophages that were histologically consistent with foamy macrophages there was a marked increase in this particular population of macrophages in ApoE^−/−^ mice as compared to C57BL/6 mice (Fig. 3a, b). Moreover, foamy macrophages represented the overwhelming majority of F4/80^+^ cells within the pannus and synovial lining in ApoE^−/−^ mice. In most of the synoviums, the foamy macrophages resided beneath the synovial lining but in some cases they were mixed within the synovial cell lining. The surface lining cells in ApoE^−/−^ synoviums also showed variable foam cell morphology. In addition to the higher level of inflammation surrounding the foamy macrophages, there was a 1.5-fold increase in the amount of picrosirius red staining, indicative of collagen deposition, in arthritic ApoE^−/−^ mice as compared to C57BL/6 mice (Fig. 4a, b). This illustrates another parallel between the pathology surrounding foamy macrophages within the synovium and those within atherosclerotic lesions. Taken together, these results indicate that ApoE^−/−^ mice demonstrate enhanced arthritis compared to C57BL/6 mice.

### ApoE deficiency alters synovial macrophages characteristics

**Fig. 5:**
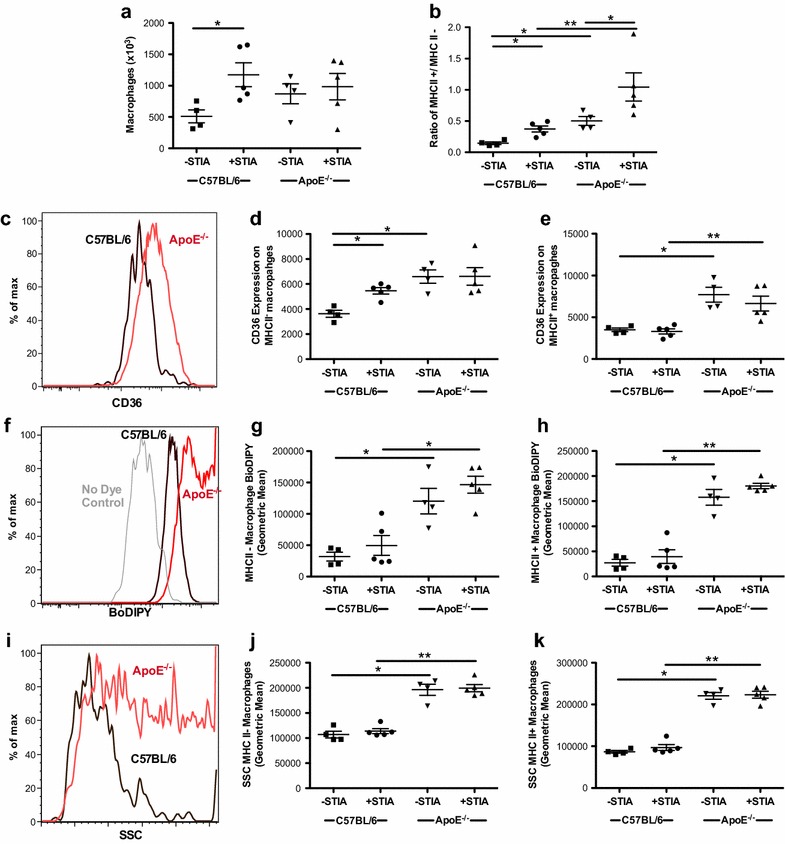
Synovial macrophages in ApoE^−/−^ mice have an MHCII^+^ phenotype and exhibit characteristics of foamy macrophages. Flow cytometric analysis of synovium from 18-week-old non-arthritic and arthritic C57BL/6 (control) and ApoE^−/−^ mice (n = 4–5 mice/group). **a** Total number of synovial macrophages. **b** The ratio of MHCII^+^ to MHC II^−^ synovial macrophages. **c** Histogram of oxidized LDL scavenger receptor, CD36, is shown with concatenation of samples from one experiment of non-arthritic control (*black*) and ApoE^−/−^ (*red*) mice. Geometric mean illustrates CD36 expression in **d** MHCII^−^ and **e** MHCII^+^ macrophages of individual mice. **f** Histogram representing uptake of lipophilic dye (BoDIPY) in a concatenation of samples from one experiment of non-arthritic control (*black*) and ApoE^−/−^ (*red*) mice. No dye control (*grey*) is included. Cumulative data displayed as geometric means in **g** MHCII^−^ and **h** MHCII^+^ macrophages. **i** Histogram of side scatter (SSC) of concatenation of samples from one experiment of non-arthritic control (*black*) and ApoE^−/−^ (*red*) mice is shown with a composite of samples illustrated as geometric mean of **j** MHCII^−^ and **k** MHCII^+^ macrophages. *Asterisk* denotes statistically significant differences: *p < 0.05, **p < 0.01

The development of acute arthritis has been shown to be associated with a progressive change in the cellular composition of immune cells within the joint, particularly macrophages [17]. We previously established our ability to identify at least two different populations of synovial macrophages (MHCII^+^ and MHCII^−^) using flow cytometry [17]. Therefore, we analyzed the impact of semi-chronic STIA on synovial macrophage composition in ApoE^−/−^ mice. Total numbers of synovial macrophages did not differ between ApoE^−/−^ and C57BL/6 mice with semi-chronic STIA (Fig. 5a). However, the higher ratio of MHCII^+^ to MHCII^−^ macrophages was more pronounced both at baseline and in the presence of semi-chronic arthritis in ApoE^−/−^ compared to C57BL/6 mice (Fig. 5b). In addition, both MHCII^+^ and MHCII^−^ synovial macrophages in ApoE^−/−^ mice displayed increased expression of CD36, a scavenger receptor that is involved in the uptake of oxidized lipoproteins (Fig. 5c–e). Both MHCII^−^ and MHCII^+^ foamy synovial macrophages exhibited a higher uptake of lipophilic dye in ApoE^−/−^ mice (Fig. 5f–h) and had higher side scatter (Fig. 5i–k), consistent with an increased accumulation of lipids within the cytoplasm.

### Arthritis does not impact atherosclerosis susceptibility in ApoE^−/−^ mice

**Fig. 6:**
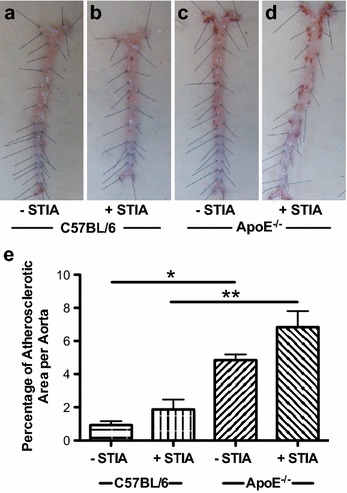
The presence of arthritis does not affect the development of atherosclerosis in ApoE^−/−^ mice. **a**–**d** Sudan IV staining of lipids (*red*) in representative aortic specimens from **a**, **b** C57BL/6 (control) and **c**, **d** ApoE^−/−^ non-arthritic (**a, c**) and arthritic mice (**b, d**). **e** Quantification of percent area of atherosclerosis (mean % ± SEM) per aorta from control (n = 4 non-arthritic and n = 5 arthritic) and ApoE^−/−^ (n = 4 non-arthritic and n = 5 arthritic) mice at 18 weeks of age. *Asterisk* denotes statistically significant differences: *p < 0.05, **p < 0.01

We have previously shown that induction of inflammatory arthritis led to the development of atherosclerosis in mice [20]. To determine if inflammatory arthritis exacerbates atherosclerosis in ApoE^−/−^ mice, a known model of hyperlipidemia-induced atherosclerosis, atherosclerotic lesion area via Sudan IV staining was investigated following STIA. Both C57BL/6 (Fig. 6a, b) and ApoE^−/−^ (Fig. 6c, d) mice exhibited a trend towards increased atherosclerosis in the presence of STIA with a 2-fold and 1.4-fold increase, respectively (Fig. 6e). When ApoE^−/−^ mice were compared to C57BL/6 there was a 5.2-fold increase in area of atherosclerosis in the absence of arthritis and a 3.6-fold increase in the area of atherosclerosis in the presence of arthritis (Fig. 6e). These data demonstrate that inflammatory arthritis has a minimal impact on the high level of atherosclerosis that is already present in ApoE^−/−^ mice on a high fat diet.

### Serum cholesterol levels are elevated in ApoE^−/−^ mice

**Fig. 7:**
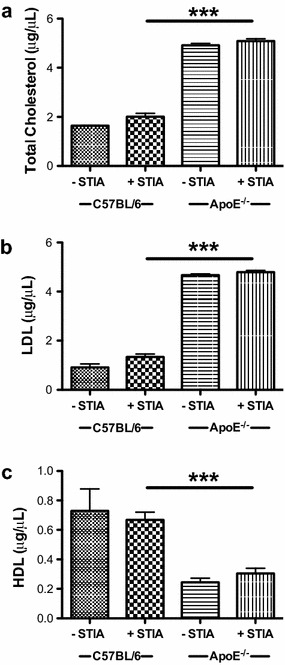
Serum cholesterol levels are elevated in ApoE^−/−^ mice and remain unchanged during semi-chronic arthritis. Serum levels of **a** TC, **b** LDL/vLDL, and **c** HDL in non-arthritic and arthritic C57BL/6 (control, n = 2 non-arthritic and n = 15 arthritic) and ApoE^−/−^ (n = 3 non-arthritic and n = 6 arthritic) mice at 4–6 months. Data are represented as mean ± SEM. *Asterisk* denotes statistically significant differences: ***p < 0.001

To determine whether induction of arthritis alters serum cholesterol profiles in ApoE^−/−^ and C57BL/6 mice, levels of TC, LDL/vLDL and HDL were measured in serum from semi-chronic STIA animals (Fig. 7). Serum levels of both TC (Fig. 7a) and LDL/vLDL (Fig. 7b) were significantly greater in arthritic ApoE^−/−^ mice (2.5-fold for TC and 3.6-fold for LDL/vLDL) compared to arthritic C57BL/6 mice. HDL levels were 38 % lower in arthritic ApoE^−/−^ mice compared to arthritic C57BL/6 animals (Fig. 7c). However, there were no significant intra-genotype differences in lipid profile upon induction of STIA. These data suggest that although baseline lipid profiles are substantially different between ApoE^−/−^ and C57BL/6 control mice, the induction of arthritis does not significantly change the lipid levels in ApoE^−/−^ mice.

### IL-6 levels are enhanced in arthritic ApoE^−/−^ mice

**Fig. 8:**
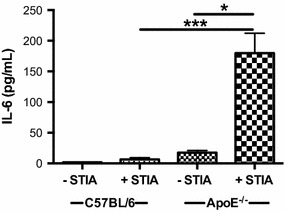
Levels of circulating IL-6 are increased in arthritic ApoE^−/−^ mice. Serum levels of IL-6 from non-arthritic and arthritic C57BL/6 (control, n = 2 non-arthritic and n = 13 arthritic) and ApoE^−/−^ (n = 3 non-arthritic and n = 6 arthritic) mice at 4–6 months. Data are represented as mean ± SEM. *Asterisk* denotes statistically significant differences. *p < 0.05, ***p < 0.001

Cytokines and chemokines are central to the development of inflammatory arthritis in both humans and mice [1]. To determine which inflammatory mediators may be responsible for the increased disease severity observed in ApoE^−/−^ mice during semi-chronic arthritis, circulating levels of cytokines and chemokines were measured in ApoE^−/−^ and C57BL/6 mice with and without arthritogenic K/BxN serum. The analysis included IL-1α, IL-1β, TNF-α, KC, MCP-1, IL-10, TGF-β, IL-4, IL-13, IL-2, IL-6, IFN-γ, IL-12p70, IL-17A, RANKL and IL-12/23p40 (Fig. 8; Additional file 3). Although the presence of arthritis in ApoE^−/−^ mice was associated with increased levels of G-CSF, KC/Gro-a and decreased levels of IL-21, IL-23 and MIP-2 (Additional file 3), the greatest change was observed in IL-6 (Fig. 8). The induction of arthritis was correlated with a 27-fold increase of serum IL-6 in arthritic ApoE^−/−^ mice compared to arthritic C57BL/6 mice (Fig. 8). As IL-6 is a known mediator of both atherosclerosis and RA, our data suggest a potential role for this pro-inflammatory cytokine in our model.

## Discussion

The role that dyslipidemia plays in the development of arthritis in murine models of RA remains controversial. Here, we utilized the semi-chronic STIA model of inflammatory arthritis since this model resembles the effector phase of RA and does not require the adaptive immune response. We show that ApoE^−/−^ mice, which spontaneously develop hyperlipidemia and atherosclerosis, also exhibit a heightened level of inflammatory arthritis when compared to control mice. This heightened inflammation is associated with an increased number of foamy macrophages in the synovium and elevated serum IL-6 levels. Nonetheless, the presence of inflammatory arthritis is not associated with changes in atherosclerosis or hyperlipidemia in ApoE^−/−^ mice. These data suggest that ApoE may play a novel suppressive role in inflammatory arthritis.

Currently, there are a plethora of therapeutics to treat dyslipidemia in RA patients. However, treatment of RA patients with atorvastatin, a cholesterol lowering medication, has been shown to decrease swollen joint count [26]. Further, previous studies using hypercholesterolemic mice to evaluate the relationship between dyslipidemia and inflammatory arthritis have yielded conflicting results. Prior studies utilized the CIA model to assess atherosclerosis and arthritis in ApoE^−/−^ mice [14, 15]. Asquith et al. found that ApoE^−/−^ animals are resistant to CIA, while Postigo et al. show that ApoE^−/−^ mice are more susceptible to chronic joint disease. Important differences in these animal models that may contribute to the discrepant results include differing genetic background (C57BL/6 mice expressing MHC Class II H-2^b^ in Asquith et al. versus B10.RIII mice expressing MHC Class II H-2^r^ in Postigo et al.) and the immunological basis of the arthritogenic trigger (anti-collagen type II (CII) antibodies in response to immunization with chicken CII in Asquith et al. and bovine CII in Postigo et al.).

We utilize an entirely different approach with the acute and semi-chronic STIA model where there is passive transfer of arthritogenic anti-glucose-6-phosphate isomerase antibodies twice a month. The arthritis that ensues in these mice has almost 100 % penetrance and the mice develop maximum arthritis within 1 week after each injection of K/BxN serum. This is in contrast to the CIA model, which has a delayed onset of disease (6–8 weeks after CII immunization) and diminished penetrance (65–75 % of control animals developing disease in the CIA models used by Asquith et al. and Postigo et al.). In addition, the semi-chronic STIA model allows us to bypass the use of adjuvant, which may complicate interpretation of the results. Moreover, this model allows us to assess the impact of semi-chronic arthritis on the development of atherosclerosis, which is a complication of longstanding disease. Here, we demonstrate that deficiency in ApoE potentiates and sustains the presence of inflammatory arthritis.

Global deletion of ApoE results in extreme elevations in serum cholesterol [27]. ApoE deficiency is known to exert effects on hematopoietic stem cell proliferation and mobilization, monocytosis and macrophage accumulation in tissue [28, 29]. In our semi-chronic STIA model, loss of ApoE is associated with changes to the properties of synovial macrophages. In our previous study using the acute STIA model (one time passive transfer glucose-6-phosphate antibodies), we demonstrated that MHCII^+^ macrophage subsets predominate during the propagation phase of arthritis while the MHCII^−^ macrophage becomes the primary cell in the resolution phase of arthritis [17]. These data suggest that ApoE^−/−^ mice may be more primed for the induction of arthritis since they exhibit a higher proportion of MHCII^+^ to MHCII^−^ macrophages. However, it is also possible that the increase in inflammation is related to a lack of resolution of inflammation.

ApoE^−/−^ mice are also able to amass foamy macrophages within the synovium. Although foamy macrophages are a well-established mediator of atherosclerotic disease, their role in the arthritic joint is not as clear. Immunohistochemical analysis of human synovial membranes reveal that foam cells are present in the synovium of RA patients [30]. In addition, a recent study observed foamy macrophages in arthritic/atherosclerotic rabbit joints [31]. Our data show increases in the number of foamy macrophages in ApoE^−/−^ mice compared to control in the semi-chronic STIA model. Interestingly this increase in foamy macrophages is associated with higher levels of inflammation in ApoE^−/−^ mice (Fig. 2) but not an increase in damage to the joint or erosive disease. However, the presence of foamy macrophages appear to be associated with enhanced collagen deposition (Fig. 4) that is reminiscent of atherosclerotic plaques and further support an extension of the pathogenic role of foamy macrophages from the vasculature to the synovium.

Our data show that ApoE^−/−^ mice have increased expression of CD36 on synovial macrophages. This is consistent with previous reports showing elevated expression of CD36 in ApoE^−/−^ peritoneal macrophages [32]. CD36 is a scavenger receptor involved in the uptake of oxLDL and the subsequent development of foamy macrophages. CD36 signaling can inhibit migration and thus promote macrophage trapping within tissue. It can also stimulate the production of inflammatory cytokines [32, 33]. It is possible that CD36 is critical for the development of arthritis in our model similar to its role in atherosclerosis where loss of CD36 improves atherosclerosis in ApoE^−/−^ mice by over 60 % [32].

The exact role of the differential expression of CD36 on MHCII^−^ and MHCII^+^ synovial macrophages is unclear. Data from an acute arthritis model suggest that the majority of tissue-resident macrophages are MHCII^−^ and are involved in attenuating arthritis as opposed to the MHCII^+^ cells that are monocyte-derived and of which a specific subpopulation is involved in propagating arthritis [17]. If these data are extrapolated to the semi-chronic arthritis model it would suggest that the potential impact of CD36 up-regulation on enhancing inflammatory arthritis is more critical in MHCII^+^ cells, which can propagate inflammation, than the MHCII^−^ cells which attenuate inflammation. Future studies will be needed to investigate the requirement for CD36 in synovial macrophages and to further characterize this heterogeneous macrophage population. These studies should evaluate not only the expression of surface markers on foamy macrophages isolated from the synovium in order to differentiate them from foamy macrophages within other cellular niches, but also look at gene expression on a single-cell basis.

Transcriptional profiling will also help further investigations into another potential target in our system, IL-6. Polymorphisms in IL-6 and serum levels of IL-6 have been shown to be associated with an increased risk of metabolic syndrome [34, 35]. In addition, studies evaluating the chronic inflammation associated with obesity and increased risk of metabolic syndrome have suggested that IL-6 is an important cytokine released by adipose tissue macrophages [36]. Our data reveal large systemic increases in IL-6 during the course of inflammatory arthritis in ApoE^−/−^ mice. This is similar to data from a French cohort showing a correlation between serum IL-6 levels and both swollen joint count and structural damage to the joint in RA patients [37]. It is also consistent with the success of anti-IL-6 receptor therapy, tocilizumab, in treating RA [38]. However, it is likely that IL-6 is a downstream product of the foamy macrophages as it is not required for the acute STIA model [39]. IL-6 may also propagate the production of foamy macrophages, as in vitro experiments have shown that IL-6 plays a role in increasing lipid uptake by macrophages [40]. Future studies will be needed to determine the requirement for IL-6 in the development of foamy macrophages and their infiltration into the joint and the ability of foamy macrophages to produce IL-6.

Although IL-6 is also an important mediator of cardiovascular disease, its augmentation in arthritic ApoE^−/−^ mice is not associated with a pronounced increase in atherosclerosis. It is possible that the systemic increase in IL-6 is not associated with local production of IL-6 in both the joint and the aorta. It may also be difficult to further enhance atherosclerosis in ApoE^−/−^ mice, which is already at a saturated level due to a combination of both genetic and dietary factors. In support of this assertion, CIA studies performed on ApoE^−/−^ mice show no change in atherosclerosis [41, 42]. Nonetheless this negative data is important to help understand the interplay between the presence of semi-chronic inflammatory arthritis and the development of atherosclerosis.

Taken together, these studies demonstrate that ApoE itself has a major effect on the development of arthritis, which may be mediated through the formation of foamy macrophages within the synovium. The identification of foamy macrophages within the inflamed arthritic joint provides a potential mechanistic link between inflammatory arthritis and atherosclerosis. Improved understanding of the connection between arthritis and atherosclerosis is crucial as we evaluate therapies that will not only improve arthritis but also decrease patients’ risk of cardiovascular disease.

## Conclusions

The present study examines the relationship between inflammatory arthritis and atherosclerosis. Our model shows that ApoE is essential for limiting the development of semi-chronic inflammatory arthritis. In contrast, the combination of inflammatory arthritis and ApoE deficiency does not enhance atherosclerosis beyond the high level observed in non-arthritic ApoE^−/−^ mice. However, our data provide additional support that factors beyond the absolute levels of lipids may be important for the development of inflammatory arthritis in ApoE^−/−^ mice. Our data also support a critical role for foamy macrophages and IL-6 production in the progression of inflammatory arthritis. Future studies may uncover the dual role for monocytes in the development of atherosclerosis and arthritis in ApoE^−/−^ mice and address the effects of ApoE deficiency on joint foam cell formation, IL-6 production and arthritis development in a cell-type specific fashion.

## Additional files

10.1186/s12967-016-0912-y Antibodies utilized for flow cytometry studies.
10.1186/s12967-016-0912-y The response to acute arthritis is similar between ApoE^−/−^ mice and C57BL/6 mice. C57BL/6 (control) and ApoE^−/−^ mice (n = 6–7 mice/group) were fed chow until 8 weeks of age, at which time they were started on a Western diet. Animals then received one injection of K/BxN serum at 12 weeks of age. Arthritis severity was scored every other day via clinical score for 2 weeks. Data are represented as mean ± SEM.
10.1186/s12967-016-0912-y Alteration of levels of circulating cytokines/chemokines in C57BL/6 and ApoE^−/−^ mice with induction of arthritis. Serum levels from non-arthritic and arthritic C57BL/6 (control, n = 2 non-arthritic and n = 13 arthritic) and ApoE^−/−^ (n = 3 non-arthritic and n = 6 arthritic) mice at 4–6 months. Data are represented as mean ± SEM. * denotes statistically significant differences. *p < 0.05, **p < 0.01,***p < 0.001.

## Abbreviations

STIA: serum transfer induced arthritis
ApoE: apolipoprotein E
RA: rheumatoid arthritis
oxLDL: oxidized low-density lipoprotein
IL-6: interleukin-6
