# Supplementary material for: ApoE deficiency exacerbates the development and sustainment of a semi-chronic K/BxN serum transfer-induced arthritis model
Source: J Transl Med. 2016 Jun 10;14:170. doi: 10.1186/s12967-016-0912-y (PMC4901400; doi:10.1186/s12967-016-0912-y)
Supplement: Supplementary file 1 — 10.1186/s12967-016-0912-y Antibodies utilized for flow cytometry studies. [file 12967_2016_912_MOESM1_ESM.docx]

**Additional File 1. Antibodies Utilized for Flow Cytometry Studies**

| **Antigen** | **Fluorochrome** | **Manufacturer** | **Clone** |
| --- | --- | --- | --- |
| CD11b | PerCPCy5.5 | eBioscience | M1/70 |
| MHC II | eFluor450 | eBioscience | M5/114.15.2 |
| CD36 | APC | BD Bioscience | No.72-1 |
| CD45 | Alexa700 | BD Bioscience | 30-F11 |
| Ly6C | APCCy7 | BD Bioscience | AL-21 |
| CD64 | PE | Biolegend | X54-5/7.1 |
| Siglec F | PECF594 | BD Bioscience | E50-2440 |
| Ly6G | PECy7 | Biolegend | 1A8 |
