# Supplementary material for: ApoE deficiency exacerbates the development and sustainment of a semi-chronic K/BxN serum transfer-induced arthritis model
Source: J Transl Med. 2016 Jun 10;14:170. doi: 10.1186/s12967-016-0912-y (PMC4901400; doi:10.1186/s12967-016-0912-y)
Supplement: Supplementary file 2 — 10.1186/s12967-016-0912-y The response to acute arthritis is similar between ApoE−/− mice and C57BL/6 mice. C57BL/6 (control) and ApoE−/− mice (n = 6–7 mice/group) were fed chow until 8 weeks of age, at which time they were started on a Western diet. Animals then received one injection of K/BxN serum at 12 weeks of age. Arthritis severity was scored every other day via clinical score for 2 weeks. Data are represented as mean ± SEM. [file 12967_2016_912_MOESM2_ESM.docx]

**Additional File 2. The response to acute arthritis is similar between ApoE^-/-^ mice and C57BL/6 mice.** C57BL/6 (control) and ApoE^-/-^ mice (n=6-7 mice/group) were fed chow until 8 weeks of age, at which time they were started on a Western diet. Animals then received 1 injection of K/BxN serum at 12 weeks of age. Arthritis severity was scored every other day via clinical score for two weeks. Data are represented as mean ± SEM.
