# Supplementary material for: ApoE deficiency exacerbates the development and sustainment of a semi-chronic K/BxN serum transfer-induced arthritis model
Source: J Transl Med. 2016 Jun 10;14:170. doi: 10.1186/s12967-016-0912-y (PMC4901400; doi:10.1186/s12967-016-0912-y)
Supplement: Supplementary file 3 — 10.1186/s12967-016-0912-y Alteration of levels of circulating cytokines/chemokines in C57BL/6 and ApoE−/− mice with induction of arthritis. Serum levels from non-arthritic and arthritic C57BL/6 (control, n = 2 non-arthritic and n = 13 arthritic) and ApoE−/− (n = 3 non-arthritic and n = 6 arthritic) mice at 4–6 months. Data are represented as mean ± SEM. * denotes statistically significant differences. *p < 0.05, **p < 0.01,***p < 0.001. [file 12967_2016_912_MOESM3_ESM.docx]

**Additional File 3. Alteration of levels of circulating cytokines/chemokines in C57BL/6 and ApoE^-/-^ mice with induction of arthritis.** Serum levels from non-arthritic and arthritic C57BL/6 (control, n=2 non-arthritic and n=13 arthritic) and ApoE^-/-^ (n=3 non-arthritic and n=6 arthritic) mice at 4-6 months. Data are represented as mean ± SEM. * denotes statistically significant differences. * p<0.05, ** p<0.01,*** p < 0.001.
